# PYCR1 promotes liver cancer cell growth and metastasis by regulating IRS1 expression through lactylation modification

**DOI:** 10.1002/ctm2.70045

**Published:** 2024-10-18

**Authors:** Haoyu Wang, Mu Xu, Tong Zhang, Jinkun Pan, Chaopu Li, Bei Pan, Linpeng Zhou, Yun Huang, Chenzi Gao, Mengping He, Yao Xue, Xuetao Ji, Xu Zhang, Ning Wang, Hongwen Zhou, Qian Wang, John Zhong Li

**Affiliations:** ^1^ The Key Laboratory of Rare Metabolic Disease Department of Biochemistry and Molecular Biology The Key Laboratory of Human Functional Genomics of Jiangsu Province Key Laboratory of Targeted Intervention of Cardiovascular Disease Collaborative Innovation Center for Cardiovascular Disease Translational Medicine Nanjing Medical University Nanjing Jiangsu China; ^2^ Department of Laboratory Medicine Nanjing First Hospital Nanjing Medical University Nanjing Jiangsu China; ^3^ School of Basic Medicine and Clinical Pharmacy Nanjing First Hospital China Pharmaceutical University Nanjing Jiangsu China; ^4^ Department of Endocrinology The First affiliated Hospital of Nanjing Medical University Nanjing Jiangsu China; ^5^ Department of Endocrinology The affiliated Huaian No.1 People's Hospital of Nanjing Medical University Northern Jiangsu Institute of Clinical Medicine Huaian Jiangsu China; ^6^ Tianjian Laboratory of Advanced Biomedical Sciences Institute of Advanced Biomedical Sciences Zhengzhou University Zhengzhou Henan China

**Keywords:** h3k18, irs1, lactylation, liver cancer, pycr1

## Abstract

**Background:**

Liver cancer (LC) is among the deadliest cancers worldwide, with existing treatments showing limited efficacy. This study aimed to elucidate the role and underlying mechanisms of pyrroline‐5‐carboxylate reductase 1 (PYCR1) as a potential therapeutic target in LC.

**Methods:**

Immunohistochemistry and Western blot were used to analyse the expression of PYCR1 in LC cells and tissues. EdU assays, colony‐forming assays, scratch wound healing assays, Transwell assays, nude mouse xenograft models and nude mouse lung metastasis models were used to detect the growth and metastasis abilities of LC cells. Transcriptome sequencing was used to search for downstream target genes regulated by PYCR1, and metabolomics was used to identify the downstream metabolites regulated by PYCR1. ChIP assays were used to analyse the enrichment of H3K18 lactylation in the IRS1 promoter region.

**Results:**

We found that the expression of PYCR1 was significantly increased in HCC and that this high expression was associated with poor prognosis in HCC patients. Knockout or inhibition of PYCR1 inhibited HCC cell proliferation, migration and invasion both in vivo and in vitro. In addition, we revealed that knocking out or inhibiting PYCR1 could inhibit glycolysis in HCC cells and reduce H3K18 lactylation of the IRS1 histone, thereby inhibiting IRS1 expression.

**Conclusions:**

Our findings identify PYCR1 as a pivotal regulator of LC progression that influences tumour cell metabolism and gene expression. By demonstrating the potential of targeting PYCR1 to inhibit LC cell proliferation and metastasis, this study identified PYCR1 as a promising therapeutic target for LC.

**Highlights:**

- Pyrroline‐5‐carboxylate reductase 1 (PYCR1) promotes the proliferation and metastasis of liver cancer (LC) cells.
- The expression of PYCR1 in LC is regulated by DNA methylation.
- Knocking down or inhibiting PYCR1 inhibits glycolysis as well as the PI3K/AKT/mTOR and MAPK/ERK pathways in LC cells.
- PYCR1 regulates the transcriptional activity of IRS1 by affecting H3K18 lactylation in its promoter region.

## Graphical abstract

Schematic diagram illustrating the mechanism by which PYCR1 knockout/inhibition hinders liver cancer (LC) progression by regulating IRS1 expression through lactylation. Upon PYCR1 knockout/inhibition, LC glycolysis is inhibited, and the intracellular lactate level decreases. As a result, the enrichment of H3K18la in the promoter region of IRS1 is lost, leading to a reduction in IRS1 expression. Immediately afterward, the downstream activation of the PI3K/AKT/mTOR and MAPK/ERK pathways was weakened, resulting in a failure to promote the growth and metastasis of LC cells.

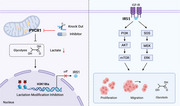

## 1 BACKGROUND

Liver cancer (LC) ranks seventh in terms of cancer incidence and third in cancer‐related mortality, imposing a significant public health challenge on a global scale.^1^, ^2^ Despite concerted efforts in the diagnosis, treatment and molecular characterisation of LC, overall recurrence and mortality rates remain high. The high mortality rate is largely attributed to the diagnosis of many patients at advanced stages, which lack effective treatment options. Recurrence and distant metastasis in advanced LC are the primary reasons for treatment failure.^3^, ^4^ Therefore, clarifying the molecular mechanisms underlying the occurrence and development of LC and identifying new diagnostic and therapeutic targets are imperative research goals.

Metabolism involves an intricate network of biochemical processes that govern energy production, biosynthesis and cellular homeostasis that play pivotal roles in tumours. Alterations in cellular metabolism, commonly referred to as metabolic reprogramming, represent a hallmark of cancer cells that enables them to sustain increased proliferation, evade apoptosis and adapt to the hostile tumour microenvironment.^5^, ^6^ Because the liver is a central metabolic organ of the body, dysregulation of metabolic pathways is not only a consequence of malignant transformation but also a driving force behind LC initiation and progression.^7^, ^8^ The functions and mechanisms of several important metabolic pathways and their corresponding genes have been elucidated in LC, like most other types of tumours. One of the best‐characterised phenotypical metabolic processes in LC is the Warburg effect, which is defined as the ability of cells to generate adenosine triphosphate (ATP) through the glycolytic pathway under aerobic conditions instead of through oxidative phosphorylation.^9^, ^10^, ^11^ GLUT1 and GLUT2, important components of the family of glucose transporters, are increased in human LC compared with adjacent tissue, and high expression levels of GLUT1 are correlated with a worse prognosis.^12^, ^13^, ^14^ Similarly, HK2, a member of the hexokinase family, has been reported to be significantly increased in LC, and its impairment negatively affects the growth and metastasis of LC.^15^, ^16^ In addition to accelerating aerobic glycolysis, a higher rate of glutamine incorporation has been revealed in LC. The glutamine transporter neutral amino acid transporter SLC1A5 is upregulated in LC cells compared with adjacent liver parenchyma cells and is significantly associated with poor survival.^17^ Furthermore, some studies reported that LC cells with CTNNB1‐activating mutations have elevated levels of glutamine synthetase and intracellular glutamine. High glutamine levels induce the activation of mTORC1 pathways and increase the sensitivity of LC cells to mTORC1 inhibitors.^18^

Exploiting the metabolic dependencies of cancer cells offers a promising avenue for the development of novel therapeutic strategies.^19^ In addition to these classic metabolic abnormalities, the role of proline metabolism in tumours has recently attracted considerable attention.^20^, ^21^ Pyrroline‐5‐carboxylate reductase 1 (PYCR1) is a key enzyme for proline synthesis that is highly expressed and plays a vital role in a variety of tumours.^22^ Kay et al. reported that PYCR1 is prominently expressed in the breast cancer (BC) stroma and in cancer‐associated fibroblasts (CAFs). Lowering the expression of PYCR1 in CAFs has been shown to effectively decrease collagen production within tumours, inhibit tumour growth and limit metastasis.^23^ Gao et al. reported that PYCR1 knockdown inhibited the proliferation, migration and invasion of lung adenocarcinoma cells by affecting the JAK/STAT signalling pathway. However, research on its function and corresponding mechanism in LC has been scarce.^24^ Here, we investigated the oncogenic role of PYCR1 in LC and clarified the corresponding molecular mechanisms, revealing its potential as a therapeutic target for LC treatment.

## 2 MATERIALS AND METHODS

### 2.1 Cell culture

The human LC cell lines HepG2 and HuH‐7 were purchased from the Typical Cultures Preservation Committee Cell Bank of the Chinese Academy of Sciences. HepG2 cells were cultured in Dulbecco's Modified Eagle Medium (DMEM) containing 10% fetal bovine serum, and HuH‐7 cells were cultured in RPMI 1640 containing 10% fetal bovine serum. All the cells were cultured at 37°C in a humidified environment with 5% CO_2_.

### 2.2 LC samples and LC tissue microarrays

LC tissue and paracancerous tissue samples were obtained from patients who underwent surgical resection at Nanjing First Hospital affiliated with Nanjing Medical University. The patients did not receive any radiotherapy or chemotherapy before surgery. The samples were pathologically confirmed and obtained with informed consent. An LC tissue microarray chip (HLivH180Su07) was purchased from Shanghai OUTDO Biotech. The array consisted of 90 paired LCs and corresponding adjacent nontumour tissues. The corresponding clinicopathological information was available for each LC tissue dot, and informed consent was obtained from each participant involved.

### 2.3 CRISPR‐Cas9 gene knockout

The sgRNA sequences targeting PYCR1 (PYCR1 sgRNA‐1‐F: CACCGCGTGCCTGTGGCATACACGG, PYCR1 sgRNA‐1‐R: AAACCCGTGTATGCCACAGGCACGC, PYCR1 sgRNA‐2‐F: CACCGGAAGTTGACACCCCACAACA and PYCR1 sgRNA‐2‐R: AAACTGTTGTGGGGTGTCAACTTCC) for CRISPR (clustered regularly interspaced short palindromic repeats)‐Cas9 gene knockout were designed using the GPP portal (https://portals.broadinstitute.org/gppx/crispick/public). Annealed sgRNA oligos were cloned and inserted into the lentiCRISPRv2 vector, which was a gift from Feng Zhang (Addgene plasmid # 52961; http://n2t.net/addgene:52961; RRID: Addgene_52961).^25^ The PYCR1 knockout plasmid was mixed with packaging plasmids and transfected into 293T cells to generate individual knockout lentiviruses following the manufacturer's protocol. After 48 h, the viral particles were harvested from the culture medium and filtered through a  .45 µm filter. The viral particles were then added to HepG2 and HuH‐7 cells along with polybrene to establish stable PYCR1 knockout cells. After 7 days of selection with puromycin, stable gene knockout cells were established. The knockout efficiency was validated through Western blot.

### 2.4 Xenograft tumour formation and in vivo metastasis assay

Five‐week‐old male BALB/c nude mice were maintained under specific pathogen‐free conditions and manipulated according to protocols approved by the Animal Care Committee of Nanjing Medical College. Wild‐type or PYCR1 knockout HepG2 cells in the logarithmic growth phase were digested with trypsin and washed twice with cold PBS to remove residual serum. The cells were subsequently resuspended and counted with PBS and adjusted to a 1 × 10^7^ cells/mL single‐cell suspension for implantation into the armpit of a nude mouse; specifically,  .2 mL of suspension (1 × 10^6^ cells) was implanted per mouse. For PYCR1 inhibitor treatment, logarithmic growth phase HepG2 cells were digested with trypsin and then inoculated into the axillae of nude mice at a density of 1 × 10^6^ cells per mouse. One week after xenotransplantation, the nude mice were randomised into two groups. Nude mice were weighed and injected with a PYCR1 inhibitor or an equal volume of dimethyl sulfoxide (DMSO) into the transplanted tumour at 50 mg/kg per treatment every 3 days for a total of four treatments. Xenografts were examined every 3 days with digital calipers, and tumour volumes were calculated via the following equation: volume = 1/2 (length × width^2^). Twenty‐one days later, the mice were sacrificed, and the volumes of the tumours were measured. For in vivo tumour metastasis assays, nude mice were randomly divided into three groups of six mice each. Logarithmic growth phase wild‐type or PYCR1 knockout HepG2 cells were digested with trypsin and resuspended in PBS to 1 × 10^7^ cells/mL, and the cell suspension was subsequently injected via the tail vein of the nude mice at 1 × 10^6^ cells/mL. Sixty days later, all the mice were euthanased. The lungs were surgically dissected, and metastatic nodules were counted. We quickly froze part of the isolated transplanted tumour in liquid nitrogen for long‐term storage and embedded part of it in paraffin for subsequent haematoxylin and eosin (HE) staining and immunohistochemical staining.

### 2.5 RNA‐seq and metabolomics

Wild‐type and PYCR1 knockout HepG2 cells were collected for RNA‐seq. Following total RNA extraction, libraries were prepared using the TruSeq Stranded mRNA Library Prep Kit (Illumina), and equimolar libraries were multiplexed and sequenced on an Illumina NextSeq 500 (with paired‐end 100 bp reads) by BGI Genomics. The ShinyGO tool was used to conduct pathway enrichment analysis with default parameters. FASTQ files were downloaded for standard analysis. For metabolomics, the collected LC cell samples were transferred to 2 mL EP tubes and dissolved in 1 mL of extraction solvent. After the addition of extraction solvent, the samples were vortexed and homogenised. The obtained solution was centrifuged, and the supernatant from each sample was transferred to another 2‐mL tube and concentrated to dryness under vacuum. The samples were dissolved in 300 µL of 2‐chlorobenzalanine solution, and the supernatants were filtered through a  .22‐µm membrane to obtain the prepared samples for LC‒MS. The metabolite extraction and measurement were performed by Genedenovo Biotechnology.

### 2.6 Statistical analysis

All the statistical analyses were performed using SPSS 24.0 SPSS and GraphPad Prism 6 (GraphPad) software. For in vitro and in vivo experiments, a *t*‐test or analysis of variance (ANOVA) was used to evaluate the differences between different groups. Univariate and multivariate Cox proportional hazards regression models were used to analyse potential factors associated with prognosis. Overall survival was estimated with the Kaplan–Meier method, and the log‐rank test was employed to evaluate differences. The in vitro experiments were subjected to replication on three separate biological replicates. All *p*‐values were two‐sided, and *p* < .05 was considered statistically significant. All data are presented as the means ± standard deviations (SDs) from at least three independent replicates.

## 3 RESULTS

### 3.1 PYCR1 is upregulated in LC, and its high expression is associated with poor prognosis in LC patients

**FIGURE 1:**
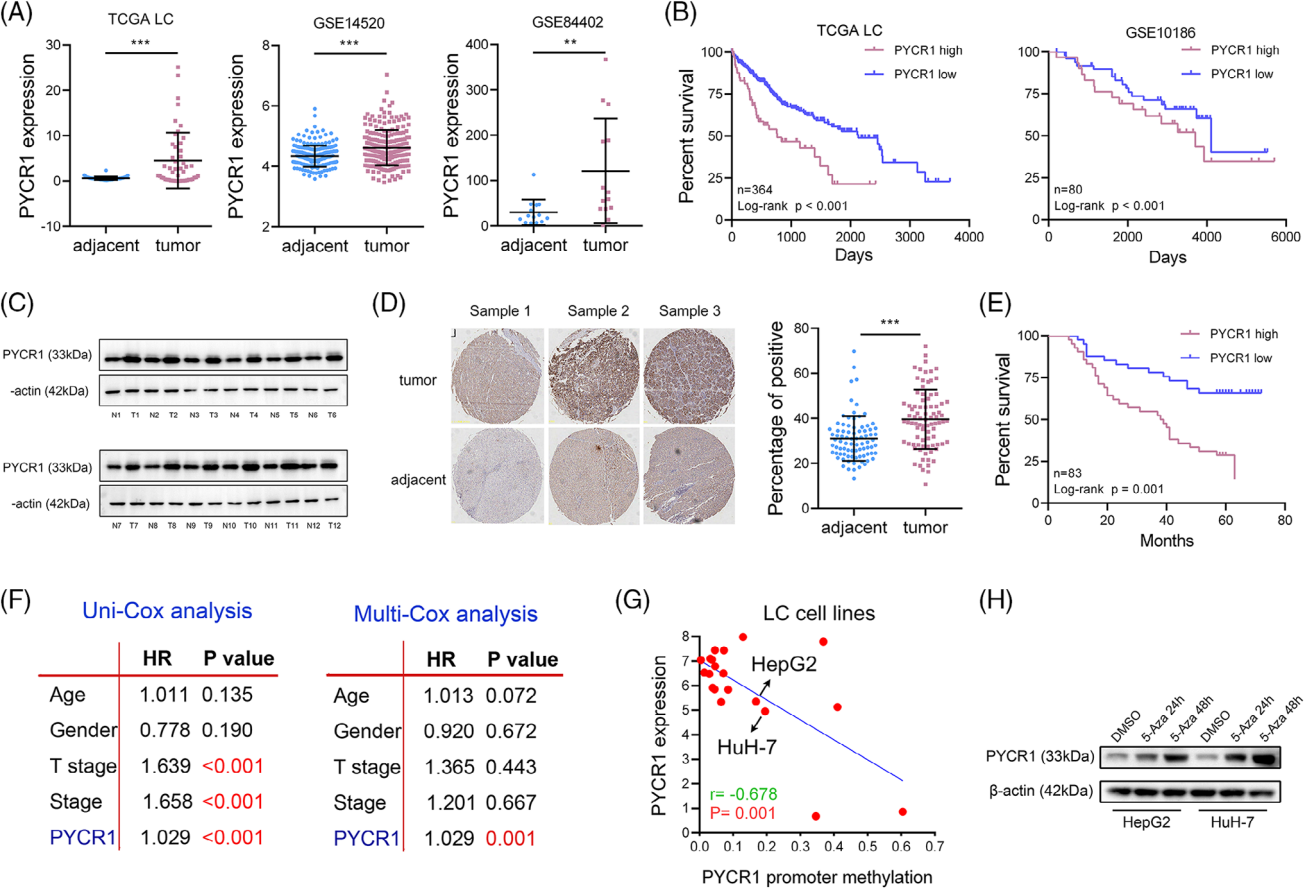
Pyrroline‐5‐carboxylate reductase 1 (PYCR1) expression is upregulated in liver cancer (LC) and high PYCR1 expression predicts poor prognosis. (A) Expression of PYCR1 in the Cancer Genome Atlas (TCGA) LC cohort, GSE14520 LC cohort and GSE84402 LC cohort. (B) Kaplan–Meier survival analysis of LC patients' overall survival based on their PYCR1 expression in the TCGA LC cohort and in the GSE10186 LC. (C) Western blot analysis of PYCR1 expression in LC and corresponding normal tissues. (D) IHC analysis of PYCR1 expression in LC and corresponding normal tissues from the tissue microarray. (E) Kaplan–Meier survival analysis of LC patients' overall survival based on their PYCR1 expression in the LC tissue microarray. (F) Uni‐Cox and Multi‐Cox survival analysis in the TCGA LC cohort. (G) Correlation analysis between the degree of the PYCR1 promoter region methylation and the PYCR1 expression in LC cell lines. (H) PYCR1 protein level in HepG2 and HuH‐7 after treating with 5‐AZA. *p < .05, **p < .01, ***p < .001.

The RNA‐seq data of LC and paracancerous tissues from the Cancer Genome Atlas (TCGA) and GEO databases were analysed. The PYCR1 RNA level was significantly increased in LC (Figure 1A). Furthermore, high expression of PYCR1 was significantly correlated with poor prognosis in LC patients (Figure 1B). Western blot analysis verified a significantly higher protein level of PYCR1 in LC tissues than in paracancerous tissues (Figure 1C). The IHC results of a tissue microarray with 83 paired LC samples also demonstrated that PYCR1 expression was significantly upregulated in LC and associated with the survival of LC patients (Figure 1D,E and Figure S1A). In addition, we found that the expression of PYCR1 is related to the pathological grade of LC and the survival status of patients (Table S1). The subsequent Cox survival analysis further supported these results (Figure 1F). We also compared the expression of PYCR1 in five LC cell lines and LO2 normal liver cells, and the results revealed that LC cell lines expressed significantly more PYCR1 than LO2 cells (Figure S1B). After analysing various LC cell lines in the Cancer Cell Line Encyclopedia (CCLE) database, we observed a negative correlation between the methylation level and the expression level of PYCR1 (Figure 1G). To verify this observation, we treated h‐cell lines with the methylation inhibitor 5‐azacytidine (5‐AZA; 10 µM), and extracted RNA and protein after 48 h of treatment. The Western blot results revealed that protein levels were significantly increased after 5‐AZA treatment (Figure 1H). Similarly, the qPCR results demonstrated that the mRNA level of PYCR1 also significantly increased after methylation inhibition (Figure S1C). These results indicate that the expression level of PYCR1 in LC is regulated by the methylation of its promoter region. In summary, the above results indicate that PYCR1 is highly expressed in LC and is associated with poor prognosis and that its expression is partly regulated by DNA methylation.

### 3.2 Knockout or inhibition of PYCR1 suppresses LC cell growth and metastasis in vitro

**FIGURE 2:**
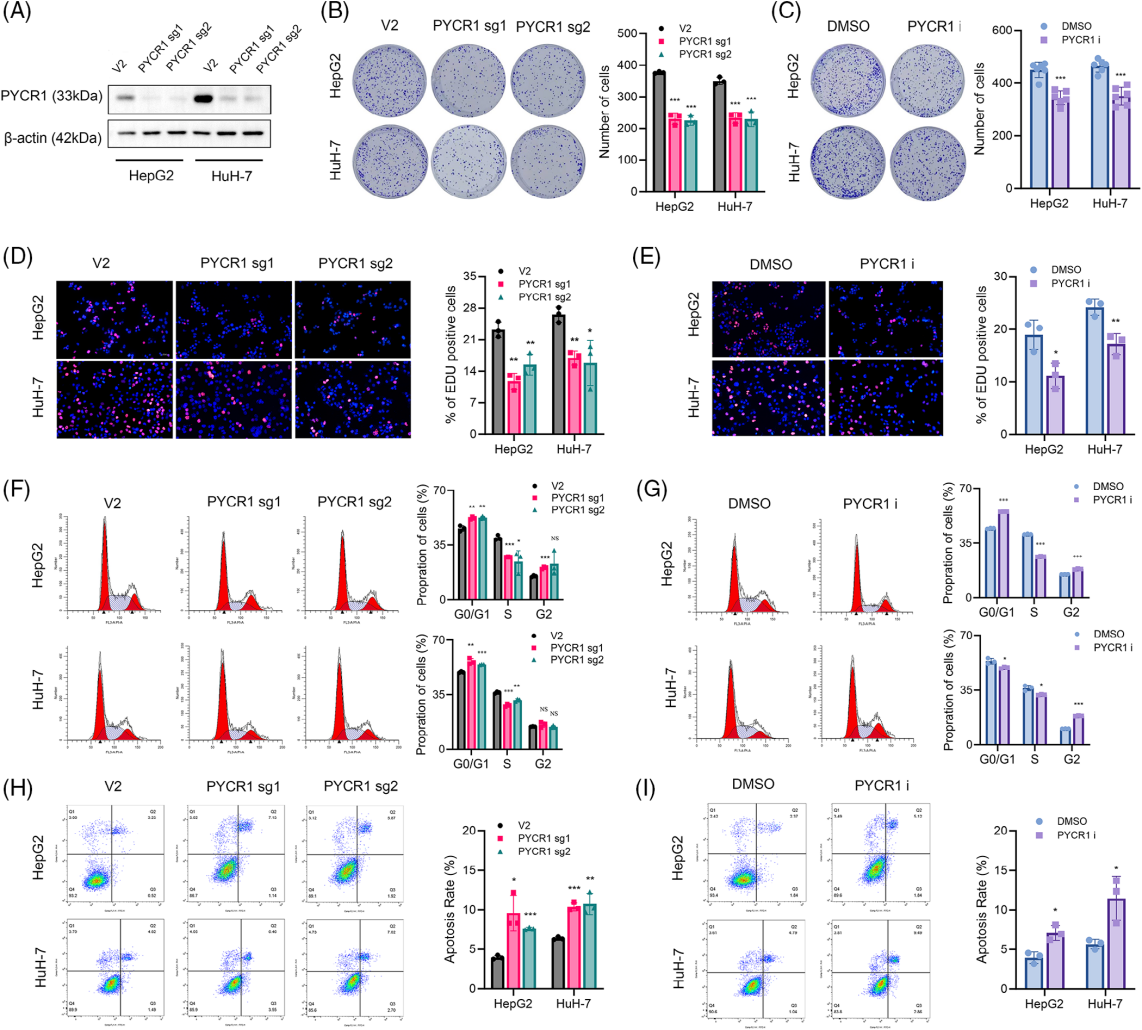
Pyrroline‐5‐carboxylate reductase 1 (PYCR1) affects liver cancer (LC) cell growth in vitro. (A) PYCR1 expression was detected in PYCR1 knockout LC cells. (B and C) HepG2 and HuH‐7 cells knocked out or inhibited PYCR1 were seeded into six‐well plates. The number of colonies was counted on the 14th day after seeding. (D and E) EdU assays were used to determine the cell proliferation ability of HepG2 and HuH‐7 cells knocked out or inhibited PYCR1. (F and G) Flow cytometric cell cycle distribution assays to detect the proportion of HepG2 and HuH‐7 cells in the G1, S and G2/M phases after PYCR1 knocked out or inhibited. (H and I) Flow cytometric cell apoptosis assays to detect cell apoptosis rate of HepG2 and HuH‐7 cells knocked out or inhibited PYCR1. *p < .05, **p < .01, ***p < .001.

CRISPR‐Cas9 gene editing technology was used to construct stable PYCR1‐knockout HepG2 and HuH‐7 cells. The knockout efficiency was verified by Western blot (Figure 2A). Moreover, we discovered an inhibitor of PYCR1 in the literature, which has been used in several studies and has demonstrated significant effects.^23^ We then commissioned the company to synthesise this inhibitor and conducted experiments with stable knockout cell lines to strengthen our results. We tested multiple concentrations of this inhibitor and found that a concentration of 20 µM significantly inhibited the proliferation of HepG2 cells at a level comparable to that of PYCR1 knockout. Because increasing the concentration did not significantly improve the inhibition rate, we selected a concentration of 20 µM for further research (Figure S1D). To validate the effects of PYCR1 on LC cells in vitro, we conducted multiple phenotyping assays. The experiments related to cell growth included CCK8 and colony formation assays. A proliferation curve generated from the absorbance data from the CCK8 experiment revealed that the proliferation rate of LC cells significantly decreased after the knockdown/inhibition of PYCR1 (Figure S2A,B). Additionally, the number of clones formed by the cells in the colony formation assay was also significantly reduced (Figure 2B,C). Because alterations in cell growth are affected mostly by the cell cycle and cell apoptosis, we next conducted EdU staining assays, flow cytometry cell cycle analyses and flow cytometry analyses of apoptosis. The results of the EdU assays demonstrated that the proportion of proliferating cells was significantly decreased after knocking out or inhibiting PYCR1 in LC cells (Figure 2D,E). In addition, flow cytometry analysis revealed a significant decrease in the proportion of S‐phase cells after the knockout/inhibition of PYCR1 (Figure 2F,G), and the apoptosis analysis revealed a significant increase in the percentage of apoptotic cells after the knockout/inhibition of PYCR1 (Figure 2H,I). These results suggest that inhibiting or knocking out PYCR1 suppressed the growth of LC cells.

We next explored the effects of PYCR1 knockout/inhibition on LC cell metastasis.

**FIGURE 3:**
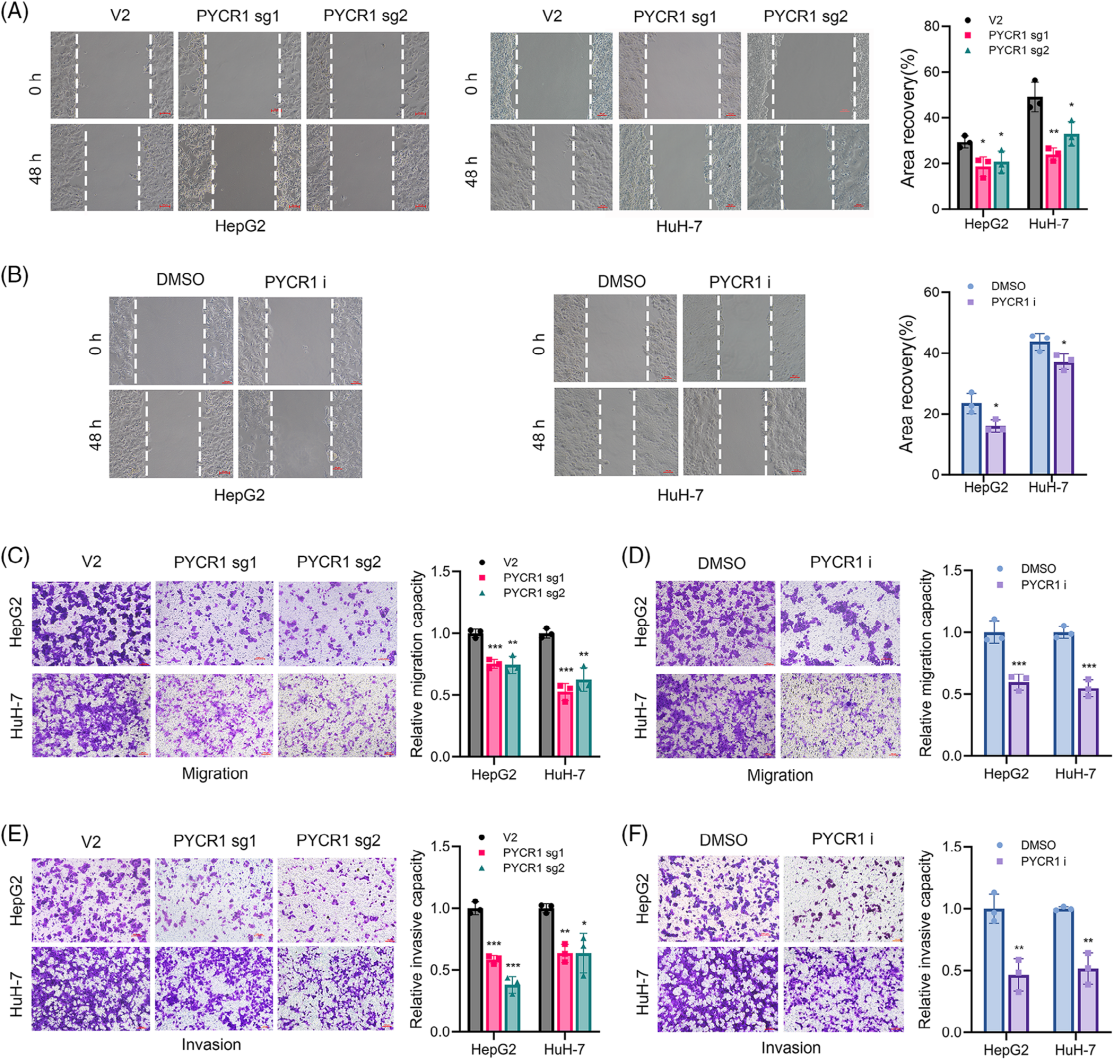
Pyrroline‐5‐carboxylate reductase 1 (PYCR1) affects liver cancer (LC) cell metastasis in vitro. (A and B) Wound healing assays were used to determine the cell migration ability of HepG2 and HuH‐7 cells knocked out or inhibited PYCR1. (C and D) Transwell assays were used to determine the migration abilities of HepG2 and HuH‐7 cells knocked out or inhibited PYCR1. (E and F) Transwell assays were used to determine the invasion abilities of HepG2 and HuH‐7 cells knocked out or inhibited PYCR1. *p < .05, **p < .01, ***p < .001.

The results of the wound healing assays revealed that the migration area of LC cells in the PYCR1 knockout or inhibition group was significantly smaller than that in the control group (Figure 3A,B). For the Transwell migration and invasion assays, the number of cells that passed through the chamber was significantly reduced upon the knockout/inhibition of PYCR1 (Figure 3C–F). These results tentatively suggest that knocking down/inhibiting PYCR1 significantly reduces the metastatic ability of LC cells.

### 3.3 Knockout or inhibition of PYCR1 hinders LC cell growth and metastasis in vivo

**FIGURE 4:**
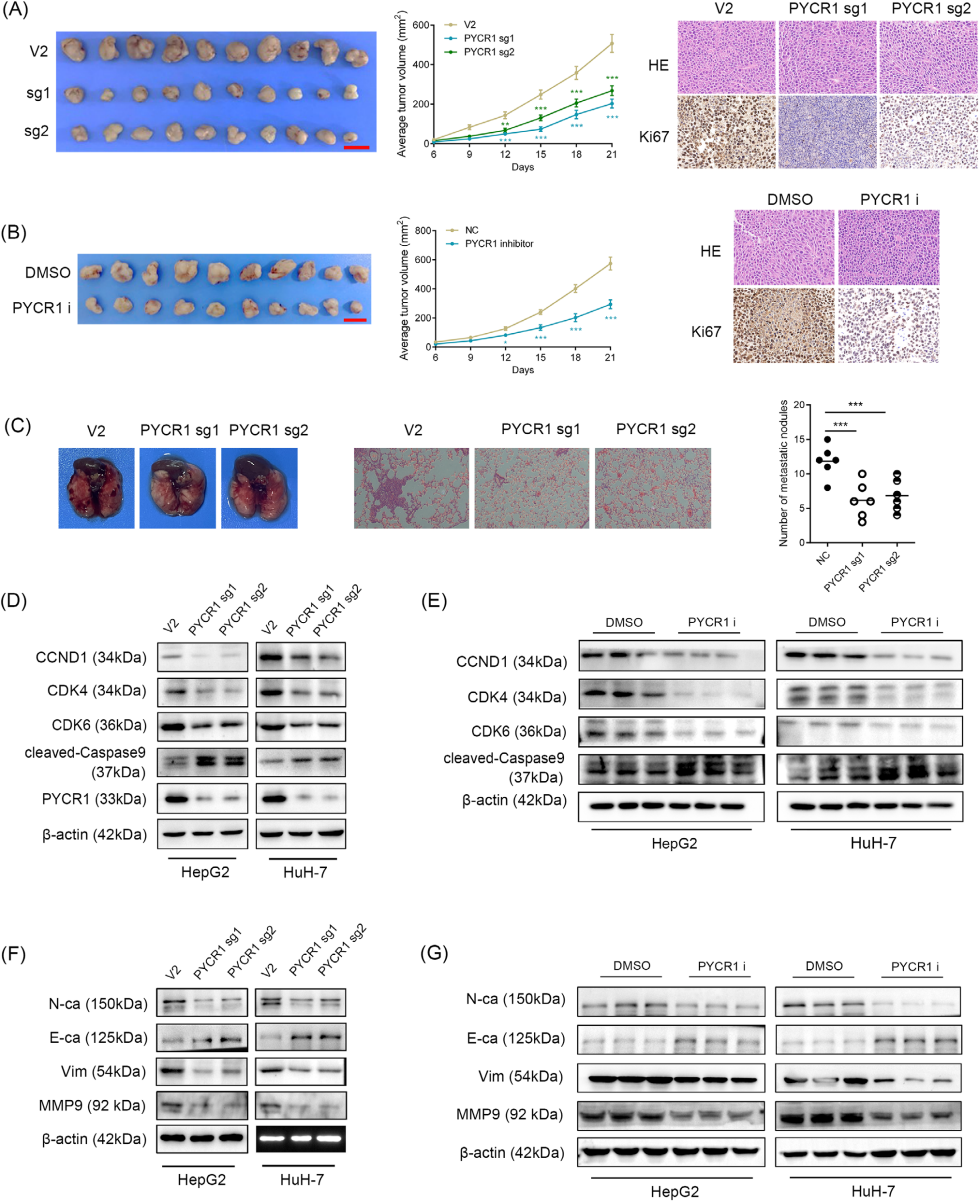
Pyrroline‐5‐carboxylate reductase 1 (PYCR1) affects liver cancer (LC) cell growth and metastasis in vivo. (A) Representative image of tumours formed in nude mice and tumour volume growth curves of the wild‐type and PYCR1 knockout group. Representative images for haematoxylin and eosin (HE) staining and Ki67 immunostaining of tumour samples from the wild‐type or PYCR1 knockout group. (B) Representative image of tumours formed in nude mice and tumour volume growth curves of the control or PYCR1 inhibitor treatment group. Representative images for HE staining and Ki67 immunostaining of tumour samples from the control or PYCR1 inhibitor treatment group. (C) Upper panel, representative images of the gross lesion in lung tissues and HE staining of metastatic nodules in the lungs from the corresponding groups. Lower panel, the statistical result of metastatic nodule numbers in the lungs from the corresponding groups. (D and E) Cell cycle‐related proteins CyclinD1, CDK4 and CDK6 and the apoptosis‐related protein cleaved‐Caspase9 detected by Western blot in HepG2 and HuH‐7 cells knocked out or inhibited PYCR1. (F and G) Tumour metastasis‐related proteins N‐cadherin, E‐cadherin, vimentin and MMP9 detected by Western blot in HepG2 and HuH‐7 cells knocked out or inhibited PYCR1. ***p < .001.

Cell phenotyping experiments can only demonstrate the role of PYCR1 in vitro. Therefore, we used a nude mouse xenograft model and a mouse model of lung metastasis via tail vein injection to explore the effects of PYCR1 knockout or inhibition on the proliferation and migration/invasion ability of LC in vivo. Using a xenograft model, we observed that tumours in the knockout group were significantly smaller than those in the control group, with a similar trend observed in the inhibitor group. In addition, we performed HE and Ki67 staining on the xenograft tissues. The results revealed that the percentage of Ki67‐positive cells was significantly reduced after knocking down or inhibiting PYCR1, which was consistent with our expectations. These results indicate that the knockdown or inhibition of PYCR1 inhibits the growth of LC cells in vivo (Figure 4A,B). In the lung metastasis model, the mice in the PYCR1 knockout group had significantly fewer lung metastasis nodules than the control mice. These findings suggest that fewer LC cells migrated from the tail vein to invade the lungs and that the migration ability of LC cells was repressed upon PYCR1 knockout in vivo (Figure 4C). To further support these results, we extracted proteins from LC cells in the knockdown and inhibitor groups. We then detected proliferation‐ and cycle‐related proteins (CCND1, CDK4 and CDK6), migration/invasion‐related proteins (N‐cadherin, E‐cadherin, vimentin and MMP9), and apoptosis‐related proteins (cleaved‐caspase 9) via Western blot. The results revealed that CCND1, CDK4, CDK6, N‐cadherin, vimentin and MMP9 expressions were significantly downregulated after PYCR1 was knocked out or inhibited. Conversely, the expression of E‐cadherin and cleaved‐caspase 9 was significantly upregulated after knocking out or inhibiting PYCR1. The results were consistent with the phenotyping experiments, further validating our conclusions (Figure 4D–G).

In summary, the knockdown/inhibition of PYCR1 in LC cells resulted in a significant decrease in cell proliferation and migration/invasion abilities, but significantly increased apoptosis. These findings support our previous findings and validate the role of highly expressed PYCR1 in promoting the malignant progression of LC.

### 3.4 Knockout or inhibition of PYCR1 perturbs the PI3K/Akt/mTOR and MAPK/ERK pathways in LC

**FIGURE 5:**
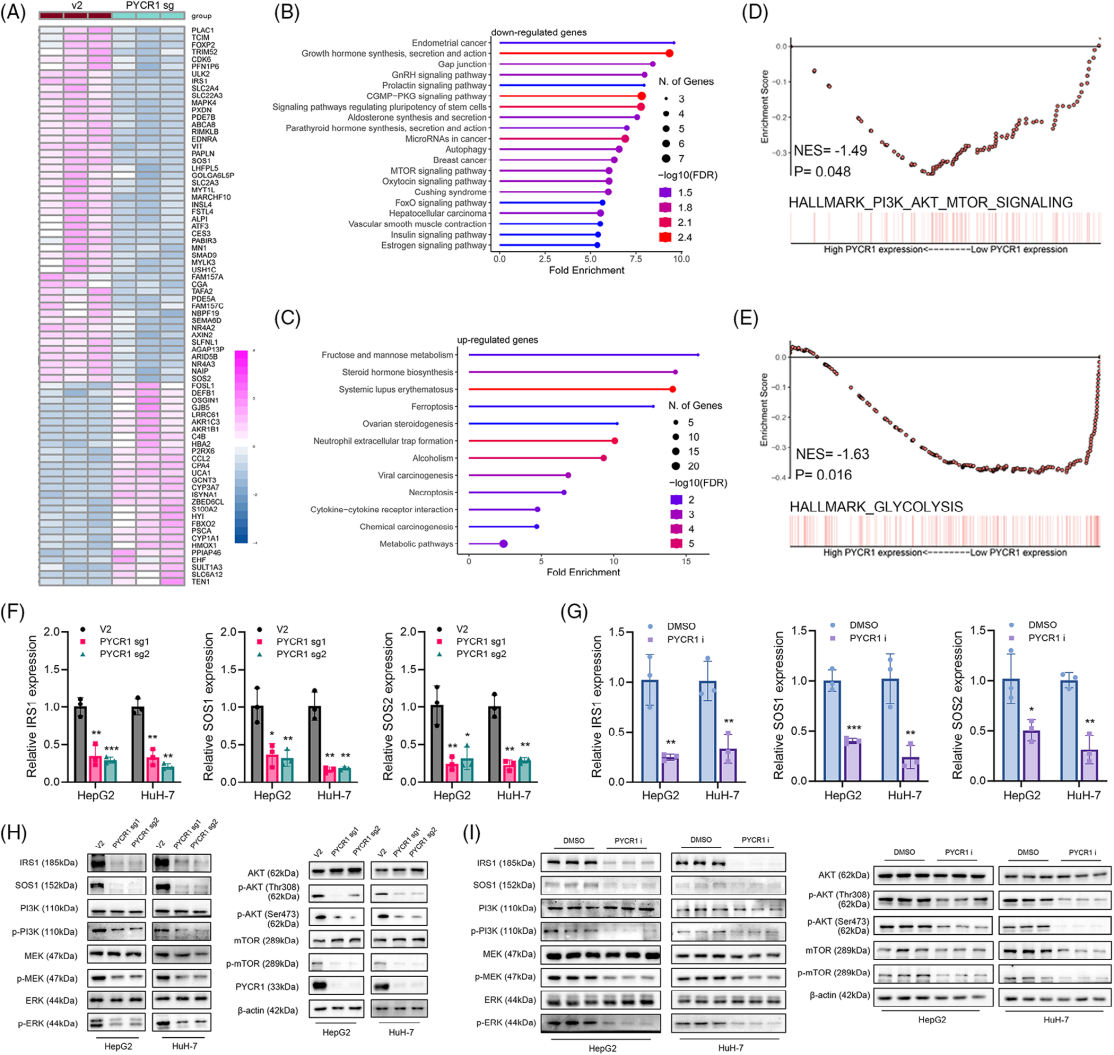
Knockout or inhibition of pyrroline‐5‐carboxylate reductase 1 (PYCR1) perturbs PI3K/Akt/mTOR and MAPK/ERK pathway in liver cancer (LC). (A) Heatmap of differently expressed genes in HepG2 cells after PYCR1 knocked out. (B and C) Pathway analysis of differential genes that are decreased (B) or increased (C) after knocking out PYCR1 in HepG2 cells. (D and E) Gene set enrichment analysis (GSEA) results are plotted to visualise the pathways related to PYCR1 in LC. (F and G) IRS1, SOS1 and SOS2 were detected by qPCR in HepG2 and HuH‐7 cells after PYCR1 knocked out or inhibited. (H and I) Functional molecules in the PI3K/Akt/mTOR and the MAPK/ERK pathway were detected by Western blot in HepG2 and HuH‐7 cells after PYCR1 knocked out or inhibited. *p < .05, **p < .01, ***p < .001.

Phenotyping experiments have shown that PYCR1 promotes the malignant progression of LC. However, the underlying mechanism remains unclear. We next used RNA‐seq to identify genes whose expression levels were significantly altered upon PYCR1 knockout in HepG2 cells (Figure 5A). Pathway enrichment analysis revealed that the genes whose expression was downregulated after PYCR1 knockout were enriched mainly in the gap junction pathway, the mTOR pathway and the insulin pathway, whereas the genes whose expression was upregulated after PYCR1 was knocked out were enriched mainly in ferroptosis and necroptosis pathways (Figure 5B,C). In addition, gene set enrichment analysis (GSEA) of RNA‐seq data from LC tissues revealed a significant positive correlation between PYCR1 expression and the PI3K/Akt/mTOR pathway (Figure 5D). Furthermore, we observed a significant positive correlation between PYCR1 expression and the vital glycolysis metabolic pathway (Figure 5E). Moreover, we found that insulin receptor substrate 1 (IRS1), an upstream gene of the PI3K/Akt/mTOR pathway, was significantly downregulated after knocking out PYCR1. Moreover, the downstream genes of IRS1, Son of Sevenless homolog 1 (SOS1) and Son of Sevenless homolog 2 (SOS2), which participate in the regulation of the MAPK/ERK pathway, were also significantly downregulated upon the knockout of PYCR1. These results were confirmed by qPCR (Figure 5F,G). To further verify the phenomena described above, proteins were extracted from PYCR1 knockout LC cells for Western blot. The results revealed that the levels of the main proteins in the PI3K/AKT/mTOR pathway and MAPK/ERK pathway were decreased (Figure 5H,I). These two pathways can strongly influence cancer progression, and their combined effects may explain the changes observed in previous phenotyping experiments.

### 3.5 Knockout or inhibition of PYCR1 impairs LC progression by regulating IRS1 expression

**FIGURE 6:**
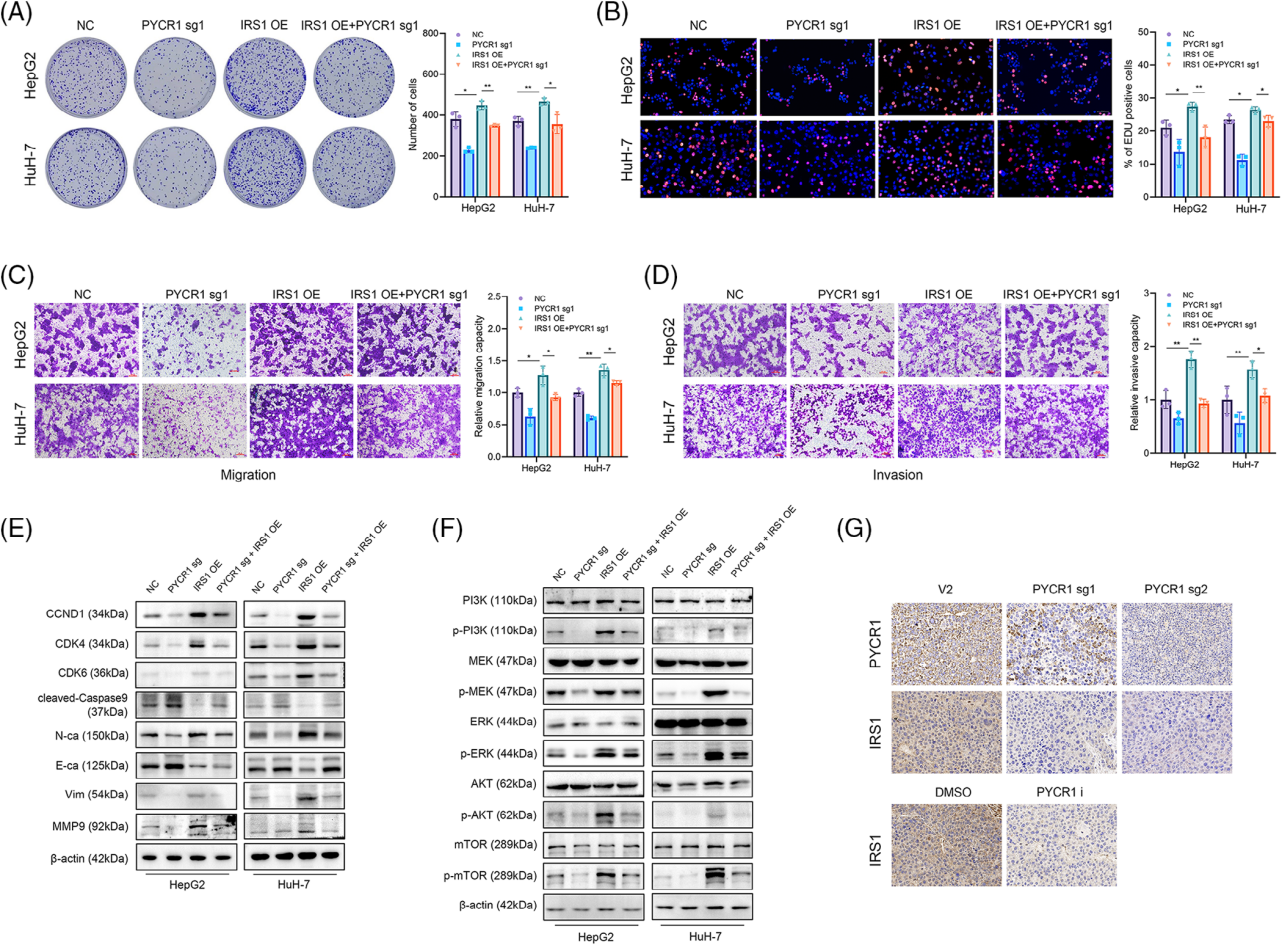
Knockout or inhibition of pyrroline‐5‐carboxylate reductase 1 (PYCR1) impair liver cancer (LC) progression depending on regulating IRS1 expression. (A) Colony formation assays demonstrated that knockout PYCR1 inhibited LC cell growth. IRS1 overexpression could abolish growth inhibition caused by PYCR1 knockout. (B) EdU assays show that IRS1 overexpression abolished the decreased proliferation rates of MDA‐MB‐231 cells caused by PYCR1 knockout. (C and D) Transwell assays demonstrated that IRS1 overexpression abolished the decreased abilities of migration and invasion caused by PYCR1 knockout. (E) Cell cycle‐related proteins, apoptosis‐related proteins and tumour metastasis‐related proteins were detected by Western blot in HepG2 and HuH‐7 cells after the corresponding treatments. (F) PI3K/Akt/mTOR pathway‐related proteins and MAPK/ERK pathway‐related proteins were detected by Western blot in HepG2 and HuH‐7 cells after the corresponding treatments. (G) Representative images of PYCR1 and IRS1 immunostaining of xenograft samples from the corresponding group. *p < .05, **p < .01.

We next explored whether PYCR1 contributes to LC progression through IRS1. PYCR1 was knocked out and IRS1 was overexpressed in LC cells, followed by a combination of the two treatments. It was hypothesised that the overexpression of IRS1 could rescue the anticancer effect caused by PYCR1 knockdown. The results of the colony formation and EdU staining assays indicated that the proliferative ability of cells was significantly improved after IRS1 was overexpressed in PYCR1‐knockout cells (Figure 6A,B). Additionally, the Transwell assay confirmed the increase in cell migration and invasion ability after IRS1 overexpression (Figure 6C,D). The Western blot results revealed that the changes in the expression of cell cycle‐related proteins, apoptosis‐related proteins and tumour metastasis‐related proteins were abolished after IRS1 was overexpressed in PYCR1‐knockout or PYCR1‐inhibited LC cells (Figure 6E). In addition, IRS1 overexpression also rescued the inhibited phosphorylation of key molecules in the PI3K/Akt/mTOR and MAPK/ERK pathways (Figure 6F). In addition, immunohistochemistry was performed on tumour tissues from previous animal experiments, confirming the downregulation of IRS1 after the knockdown/inhibition of PYCR1 in vivo (Figure 6G). These findings suggest that the impairment of LC progression resulting from the knockdown/inhibition of PYCR1 is due to the repression of IRS1.

### 3.6 PYCR1 affects IRS1 expression by regulating H3K18 lactylation in the IRS1 promoter region

**FIGURE 7:**
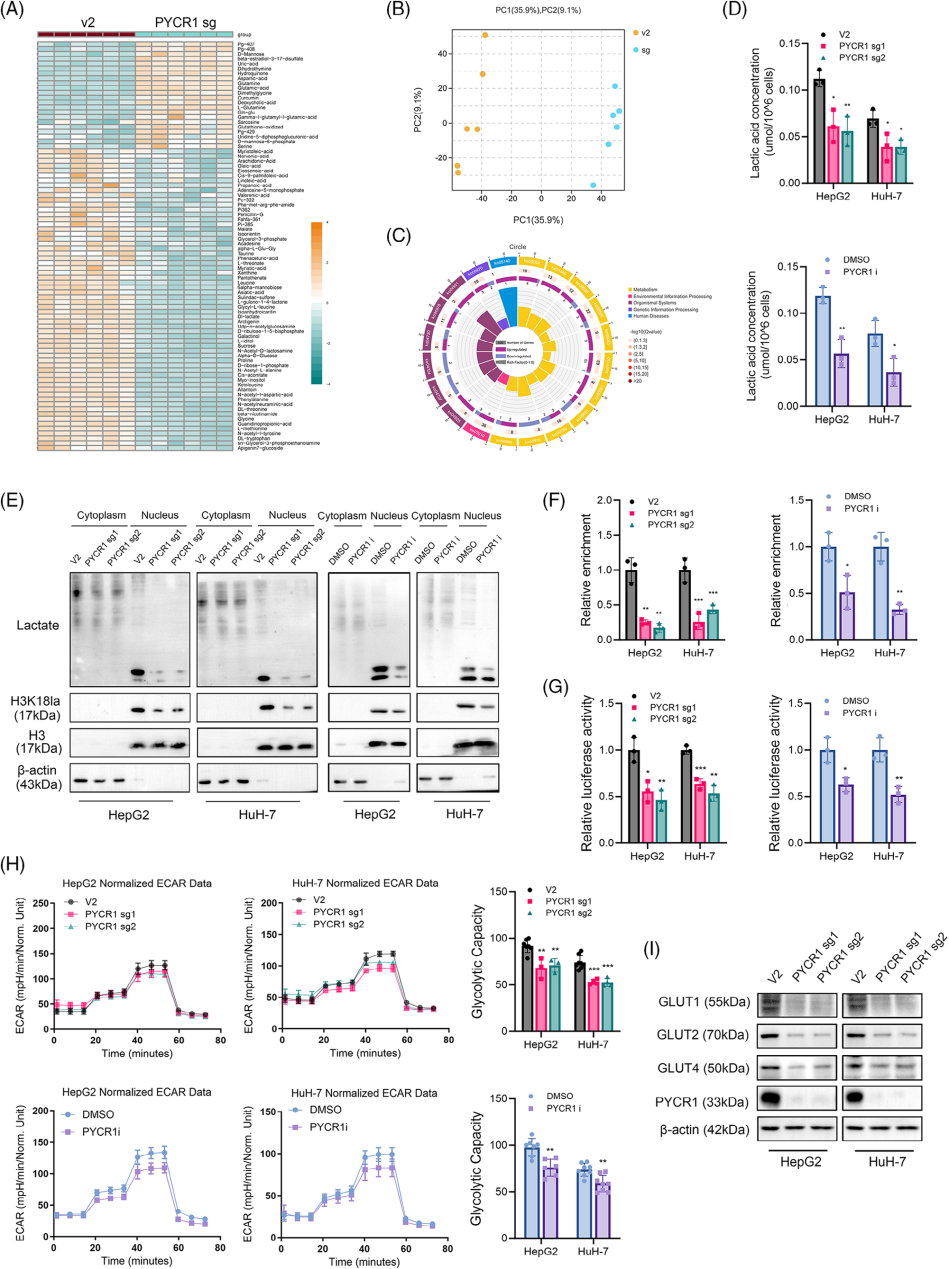
Knockout or inhibition of pyrroline‐5‐carboxylate reductase 1 (PYCR1) suppresses liver cancer (LC) cell glycolysis and further downregulates IRS1 expression through erasing H3K18 lactylation in the IRS1 promoter region. (A) Heatmap of metabolites altered in HepG2 cells after PYCR1 knocked out. (B) PCA analysis of samples for metabolomic. (C) Pathway enrichment analysis of differential metabolites in HepG2 cells after PYCR1 knocked out. (D) Lactate concentration detection in HepG2 and HuH‐7 cells after PYCR1 knocked out or inhibited. (E) Overall protein lactation level and H3K18 lactation level were detected by Western blot in HepG2 and HuH‐7 cells after PYCR1 knocked out or inhibited. (F) ChIP‐PCR assays were used to detect H3K18la enrichment in the IRS1 promoter region. (G) Luciferase reporter assays to analyse the transcriptional activity of IRS1 in HepG2 and HuH‐7 cells after PYCR1 knocked out or inhibited. (H) The extracellular acidification rate (ECAR) was measured in HepG2 and HuH‐7 cells after PYCR1 knocked out or inhibited to reflect cellular glycolysis ability. (I) Glycolysis‐related proteins were detected by Western blot in HepG2 and HuH‐7 cells after PYCR1 knocked out or inhibited. *p < .05, **p < .01, ***p < .001.

Although we determined that PYCR1 promotes LC progression through IRS1, the mechanism linking these two molecules remains unknown. PYCR1 is located primarily in mitochondria, whereas IRS1 is found in the plasma. These organelles are not in direct contact with each other, making interaction difficult. Our IP and mass spectrometry results also ruled out the possibility of an interaction between the two (not shown). Because PYCR1 is a key enzyme for proline synthesis, we explored whether the inhibitory effect of PYCR1 knockout on LC was due to a reduction in proline content. However, the results of the CCK8 and Transwell assays revealed that proline supplementation did not weaken the inhibitory effect of PYCR1 knockdown on LC (Figure S2C–E). In addition, metabolomics analyses revealed a significant difference between the control and PYCR1 knockout groups, but no notable pathway enrichment was found in the pathway analysis. However, we found that the content of lactate, a metabolite of glycolysis, was significantly reduced in the PYCR1‐knockdown group (Figure 7A–C). A significant decrease in lactate content was also observed when a biochemical kit was used (Figure 7D). This phenomenon is consistent with previous pathway enrichment results in LC tissues, in which PYCR1 expression was significantly correlated with the glycolysis pathway. Recent studies have revealed that lactate can dominate the lactylation of proteins and that one of these modifications occurs at the histone H3 lysine 18 lactylation locus (H3K18la), which can activate gene transcription.^26^, ^27^ Therefore, we hypothesised that the downregulation of IRS1 was caused by a decrease in the intracellular lactate content, which in turn caused the downregulation of histone H3K18 lactylation. To verify our speculation, we first analysed overall protein lactylation using Western blot. The results revealed that both the overall cytoplasmic protein lactylation level and histone H3K18 locus enrichment were downregulated when PYCR1 was knocked down or inhibited (Figure 7E). We then conducted ChIP‒PCR assays to detect H3K18la enrichment in the IRS1 promoter region. The results revealed that after PYCR1 knockout, H3K18la enrichment in the IRS1 promoter region was significantly reduced (Figure 7F). In addition, the results of the IRS1 promoter luciferase reporter assays revealed that the transcriptional activity of IRS1 was significantly reduced in response to PYCR1 (Figure 7G). Finally, we supplemented PYCR1 knockout LC cells with lactate and found that the suppressed IRS1 expression was significantly restored. The effect of lactate on IRS1 transcriptional activity was further verified (Figure S3A).

### 3.7 Knockout or inhibition of PYCR1 suppresses LC cell glycolysis

Because lactate is the primary product of glycolysis, we next explored whether the decrease in lactate was due to the inhibition of glycolytic function. The results of the Seahorse cell metabolic assays revealed a significant decrease in the degree of glycolysis following the inhibition/knockdown of PYCR1 in LC cells (Figure 7H). We then examined the expression of three key glycolysis enzymes HK2, PKM2, PFKFB3 and LDHA, after PYCR1 was knocked out or inhibited in LC cells. The results revealed that the expression of these genes was significantly downregulated after PYCR1 was knocked out or inhibited. In addition, we also observed the same results in xenograft tumour tissue (Figure S3B–D). Western blot analysis also confirmed a decrease in the expression of glucose uptake‐related proteins (GLUT2/3/4) after PYCR1 knockout (Figure 7I). GLUT4 expression was significantly downregulated both in the cytoplasm and membrane (Figure S3E). In addition, glucose uptake assays confirmed that glucose uptake in LC cells was reduced after PYCR1 knockout (Figure S3F). Interestingly, a significant decrease in the level of mitochondrial oxidative phosphorylation was also observed (Figure S4A). Moreover, the Western blot results indicated that the knockdown/inhibition of PYCR1 in LC resulted in changes in the levels of a series of genes related to oxidative phosphorylation (Figure S4B,C). This finding may be attributable to the localisation of PYCR1 in mitochondria and impaired mitochondrial function after the knockdown/inhibition of PYCR1. Taken together, these results suggest that knockout or inhibition of PYCR1 perturbs glucose‐related metabolic processes in LC cells, and the downregulation of IRS1 upon PYCR1 knockout or inhibition is caused by the increase in H3K18 lactylation induced by a decrease in the intracellular lactate content.

Schematic diagram illustrates the mechanism by which PYCR1 knockout/inhibition hinders LC progression. After PYCR1 knockout/inhibition, LC glycolysis is inhibited, and the intracellular lactate level decreases. As a result, the enrichment of H3K18la in the promoter region of IRS1 is lost, leading to a reduction in IRS1 expression. Immediately afterward, as downstream targets, PI3K/AKT/mTOR and MAPK/ERK pathway activity is weakened, resulting in a failure to promote the growth and metastasis of LC cells.

## 4 DISCUSSION

Abnormal metabolism plays a vital role in the occurrence and development of cancer. Tumour cells are often metabolically active to meet the material and energy required for their unlimited proliferation and high ability to metastasise.^5^ Given the crucial role of the liver in various metabolic pathways, studies have demonstrated that the metabolism of LC cells is highly dysregulated. In LC, metabolic processes ranging from glucose metabolism and energy generation in the form of ATP to amino acid and fatty acid metabolism undergo notable alterations.^7^, ^9^, ^28^ In addition to vigorous metabolism, the expression of many metabolic enzymes increases abnormally. Some of these abnormally increased metabolic enzymes are essential for tumour survival. These genes are important potential targets for tumour diagnosis and treatment and deserve in‐depth study.^29^

Previous studies have shown that proline metabolism is widely rewired during cancer development.^20^, ^21^ Pyrroline 5‐carboxylate reductase 1 (PYCR1) is an essential enzyme for proline synthesis that is abundantly expressed and acts as an oncogene in a variety of tumours. In BC, PYCR1‐synthesised proline maintains BC cell stemness by activating cGMP‐PKG signalling.^30^ In colorectal cancer (CRC), PYCR1 is phosphorylated at Tyr‐135 by nuclear IGF1R under hypoxia, which promotes its binding to ELK4 and recruitment to gene promoters. This phosphorylation is essential for ELK4‐Sirt7‐mediated transcriptional repression, sustaining CRC cell growth.^31^ However, the role of PYCR1 in LC has not been well studied.

Our study revealed that PYCR1 was significantly increased in patients with LC and that its elevation was associated with poor prognosis in patients with LC. Furthermore, we identified PYCR1 as an essential gene for sustaining LC cell growth and metastasis. Genetic editing to knockout PYCR1 or inhibiting its activity with an inhibitor suppressed the progression of LC both in vitro and in vivo. In terms of mechanism, RNA‐seq analysis revealed that PYCR1 knockout significantly inhibited the AKT pathway and glycolysis pathway by suppressing IRS1 expression. We subsequently confirmed these phenomena with a series of validation experiments. IRS1 is the initial member of the insulin receptor substrate family and serves as a vital intermediary between the insulin receptor and insulin‐like growth factor receptor within the insulin signalling cascade.^32^, ^33^ The activation of IRS1 modulates several important signalling pathways, such as the PI3K and MAPK signalling pathways, which participate in regulating cellular metabolism and the mitotic pathway.^34^, ^35^, ^36^ Extensive research has shown that IRS1 is involved in the occurrence and development of various cancers, including LC.^37^ Gao et al. revealed that IRS1 was significantly upregulated in patients with LC, and after controlling for age, sex and HBV infection, IRS1 expression was identified as an independent diagnostic biomarker of LC.^38^ Sakurai et al. reported that liver‐specific IRS1 knockout suppressed diethyl nitrosamine‐induced LC development in mice, which was accompanied by reduced cancer cell proliferation and reduced activation of AKT.^39^ Our study also demonstrated that IRS1 promoted the malignant progression of LC. In addition, we demonstrated that IRS1 expression was regulated by PYCR1 and that its downregulation mediated the inactivation of the AKT and MAPK pathways caused by PYCR1 knockout. We then explored the molecular mechanism by which PYCR1 regulates IRS1. Metabolomic analysis showed that the lactate level in LC cells was significantly reduced after PYCR1 knockout. Combined with the RNA‐seq results, we speculated that inhibiting PYCR1 reduces the glycolytic ability of LC cells, thereby affecting lactate production. This speculation was confirmed by subsequent Seahorse assays. Cancer cells, including LC cells, exhibit the Warburg effect; they favour glycolysis over oxidative phosphorylation, even in the presence of oxygen.^40^ This shift supports rapid cell proliferation by providing both energy and biosynthetic precursors. LC cells often overexpress key glycolytic enzymes, such as hexokinase 2 (HK2), pyruvate kinase M2 (PKM2) and lactate dehydrogenase A (LDHA), which increases glycolytic flux and contributes to their aggressive behaviour.^41^ Oncogenic signalling pathways, including the PI3K/AKT/mTOR and MYC pathways, are frequently activated in LC, where they promote glycolysis by upregulating the expression of glycolytic enzymes and glucose transporters. Elevated lactate production from enhanced glycolysis acidifies the tumour microenvironment, which promotes invasion, metastasis and immune evasion.^42^, ^43^ Recent studies have shown that lactate in cells can induce the lactylation of proteins, and histone lactylation has been revealed as a novel histone posttranslational modification that links cellular metabolism to epigenetic regulation.^27^, ^44^ Among these changes, H3 lysine 18 lactylation (H3K18la) is widely reported to be a histone marker of gene transcription activation.^26^ Through ChIP‒PCR and luciferase reporter assays, we found that knocking out or inhibiting PYCR1 resulted in a loss of H3K18la in the promoter region of IRS1 and that the transcriptional activity of IRS1 was reduced. Therefore, we believe that decreased lactate production upon PYCR1 inhibition resulted in histone H3 lysine 18 lactylation (H3K18la)‐mediated IRS1 downregulation.

In summary, our study revealed that PYCR1 is significantly increased in LC and is associated with poor patient prognosis, suggesting that PYCR1 is crucial for sustaining LC cell growth and metastasis. Mechanistically, PYCR1 knockout/inhibition disrupted the AKT and MAPK pathways by suppressing IRS1 expression. Furthermore, the molecular mechanism underlying the regulation of IRS1 by PYCR1 was explored, which showed that this process involves a reduction in lactate production and subsequent histone H3 lysine 18 lactylation (H3K18la)‐mediated transcriptional inhibition. Our research opens new avenues for the treatment of LC. These findings underscore the potential of targeting PYCR1 as a novel therapeutic strategy for treating LC. Additionally, the discovery of the molecular link between PYCR1 and IRS1 provides a valuable framework for understanding metabolic and epigenetic alterations in cancer cells. Future studies could delve deeper into the precise mechanisms by which PYCR1 and its metabolic products, such as lactate, regulate gene expression and cancer cell behaviour. Moreover, exploring the clinical relevance of PYCR1 and IRS1 in LC patient populations may offer insights into personalised medicine approaches. Overall, this research offers a promising direction for the development of novel therapeutic interventions for LC.

## 5 CONCLUSION

Our study reveals the expression pattern and tumour‐promoting role of PYCR1 in LC and identifies the downstream target IRS1 through which PYCR1 promotes LC progression. In addition, our study demonstrated that PYCR1 can regulate lactate metabolism in LC via histone lactylation to affect gene expression. Our study provides new avenues to identify novel therapeutic targets for treating LC.

## AUTHOR CONTRIBUTIONS

**Haoyu Wang**; **Mu Xu** and **John Zhong Li**: designed the experiments. **Haoyu Wang; Mu Xu; Tong Zhang; Jinkun Pan; Chaopu Li; Linpeng Zhou; Yun Huang; Chenzi Gao**: performed the experiments. **Haoyu Wang; Mu Xu; Tong Zhang; Jinkun Pan; Chaopu Li; Bei Pan; Mengping He; Yao Xue**: analysed the data. **Haoyu Wang; Mu Xu; Xu Zhang; Xuetao Ji; Ning Wang; Hongwen Zhou; Qian Wang; John Zhong Li**: contributed to discussion and wrote the manuscript. All authors approved the final manuscript.

## CONFLICT OF INTEREST STATEMENT

The authors declare that they have no conflicts of interest.

## ETHICS STATEMENT

The research was approved by the Ethics Committee of Nanjing Medical University.

## Supporting information



Supporting Information

## Data Availability

The datasets used and/or analysed during the current study are available from the corresponding author upon reasonable request.
