# Supplementary material for: PYCR1 promotes liver cancer cell growth and metastasis by regulating IRS1 expression through lactylation modification
Source: Clin Transl Med. 2024 Oct 18;14(10):e70045. doi: 10.1002/ctm2.70045 (PMC11488319; doi:10.1002/ctm2.70045)
Supplement: Supplementary file 1 — Supporting Information [file CTM2-14-e70045-s001.docx]

**Detailed Methods**

**PYCR1 inhibitor**

The inhibitor of PYCR1 was synthesized according to the route reported in a previous literature[1], and the purity of the synthesized product was verified by mass spectrometry (Figure S5). The inhibitor was diluted with DMSO, and the concentration used to treat the LC cells was 20 μM.

**RNA extraction and qPCR**

Total RNA was extracted from cells and tissues by using TRIzol reagent (Invitrogen, USA) in accordance with the manufacturer’s protocol. Reverse transcription was performed using the HiScript qRT SuperMix Kit (Vazyme, China). qPCR was conducted using an ABI 7500 real-time PCR system (Applied Biosystems, USA) with the ChamQ SYBR Color qPCR Master Mix (Vazyme, China). Fold changes were analyzed using the 2-ΔΔCT method and normalized to ACTB. The primer sequences are shown in Table S1.

**Western blot**

Total proteins were extracted from cells or tissues using lysis buffer (containing proteinase and phosphatase inhibitors). The total protein concentration in the lysate was determined using the BCA method. Protein samples (20 μg) were separated by sodium dodecyl sulfate‒polyacrylamide gel electrophoresis on separating gels using a vertical electrophoresis apparatus. After separation, the proteins in the gels were transferred to PVDF membranes and then incubated with the primary antibody at 4 °C overnight. The membrane was subsequently incubated with secondary antibodies at room temperature for 2 hours. After washing, the protein bands were detected using enhanced chemiluminescent detection reagent. Details of the antibodies used and their dilutions are listed in Supplementary Table S3.

**Immunohistochemistry**

Tissues were collected and embedded in paraffin. The section thickness was 4 μm. After being treated with fractionated ethanol and distilled water, the sections were treated with methanol and 3% H_2_O_2_ for 30 min to prevent the activity of endogenous peroxidase. The sections were washed twice with phosphate-buffered saline for 5 min each and incubated with 10% goat serum for 30 min to prevent the binding of nonspecific antibodies. After washing, the sections were incubated with primary antibodies at 4 °C overnight, followed by incubation with secondary antibodies. According to the manufacturer's protocol, the sections were stained with diaminobenzidine, sealed and visualized under a microscope. The same procedures were used for the LC tissue microarrays. Details of the antibodies used and their dilutions are listed in Supplementary Table S3.

**CCK8 assay**

A cell growth assay was performed using a CCK-8 assay kit (Beyotime, China). LC cells subjected to different treatments were inoculated into 96-well plates with 100 μL of medium. At the detection time points, 10% CCK-8 solution was added to each well, and the samples were incubated for 1–4 h in an incubator. The absorbance value at 450 nm was subsequently measured to calculate the cell viability.

**Colony formation assay**

Different experimentally treated LC cells were digested using a medium containing 0.25% EDTA, and then added to the corresponding medium and blown up as a single cell suspension. The cells were counted to adjusted to 1×10^3^ cells/ml, and added into 6-well plates with 1×10^3^ cells per well. 6-well plates were then put into 37 °C, 5% CO_2_ incubator for 10 days, the liquid was changed every three days. Once the individual cells were divided into clusters, LC cells were gently washed with PBS for 2-3 times after the medium was discarded. 1 ml of methanol was added to each well to fix the cells for 10 min, discard and dry the residual liquid at room temperature. 5% crystal violet staining solution (Beyotime, China) was added to completely cover the cells, and the cells were stained for 15 min, followed by gentle washing with PBS, and the number of cell colonies formed was observed and the clone formation rate was calculated.

**EdU assay**

EdU assays were conducted using the Cell-Light EdU DNA Cell Proliferation Kit (RiboBio, China). Briefly, LC cells were seeded into 24-well plates and cultured overnight. On the following day, the medium in the wells was discarded and EdU solution (Reagent A) was added with complete medium diluted to 50 uM EdU solution, 200 µl per well. The plates were incubated for 2 h at 37 ℃ in a thermostatic incubator, and then fixed in 4% paraformaldehyde for 30 minutes at room temperature following treatment with 2 mg/ml glycine for 5 min and 0.5% Trion X-100 for 10min. After washing three times with PBS, configure Apollo Staining Reaction Solution (per 500 µl) according to the following ratio: 25 µl Apollo® Reaction Buffer + 5 µl Apollo® Catalyst Solution + 1.5 µl Apollo® Fluorescent Dye Solution + 5 mg Apollo® Buffer Additive + 469 µl deionized water. 200 µl of staining reaction solution was added to each well and incubated at room temperature for 30 min under light-avoidance conditions. After discarding the staining solution, the wells were washed three times for 10 min each with 200 µl of PBS containing 0.5% TritonX-100, protected from light. Finally, cells were stained with Hoechst 33342 (5 μg/mL) for 30 min, washed three times with PBS and then blocked to be observed under a fluorescence microscope.The EdU incorporation rate was expressed as the ratio of EdU positive cells (red cells) to total Hoechst33342 positive cells (blue cells).

**Wound Healing Assay**

For the wounding-healing assay, LC cells subjected to different treatments were seeded into 6-well culture plates and incubated for 24 h. When the cell confluence reached 95%, a scratch wound was created with a 10 μl tip, and the well was then placed under a microscope to take a 0-hour photograph. The cells were subsequently incubated in a constant-temperature incubator. The samples were observed and photographed after 48 hours. The migration index was evaluated based on the wound closure rate.

**Transwell assay**

LC cells in 200 μl of serum-free medium were plated in the upper chamber of the Transwell system, which was coated with (invasion assay) or without (migration assay) the Matrigel mixture (BD, USA) following the manufacturer’s instructions. Then, the cells were incubated in the lower chamber with 500 μl of medium containing FBS. After 24 h of incubation, the cells that invaded or migrated into the lower chamber were fixed with 4% paraformaldehyde and stained with crystal violet for 15 min. Then, the cells were dried and photographed.

**Flow cytometry**

Apoptosis was assessed using the Annexin V-FITC Apoptosis Detection Kit (KeyGEN, China) according to the manufacturer’s protocol. Briefly, LC cells subjected to different treatments were collected and washed twice with cold PBS, followed by incubation with Annexin V-FITC and propidium iodide in the dark for 15 min at room temperature. The cell apoptosis data were analyzed with a FACSCalibur flow cytometer (BD, USA). For cell cycle detection, LC cells were washed with cold PBS, fixed in 70% ethanol for 24 hours and stained with propidium iodide for 30 minutes. Finally, the cell cycle data were analyzed via ModFit LT software (Verity, USA).

**Lactate content detection**

The lactate content was detected using LA assay kit (Solarbio, China). LC cells subjected to different treatments were cultured to 80% confluence and then harvested. After the cells were lysed with ultrasound, the supernatant was collected following centrifugation. Lactate standard solutions were diluted to create a standard curve, and the sample content was determined based on this curve. All the experiments were conducted at least three times to ensure accuracy and reliability.

**Chromatin immunoprecipitation assay**

ChIP assays were performed using the Simple ChIP Enzymatic Chromatin IP Kit (Cell Signaling Technology, USA) according to the manufacturer’s instructions with slight modifications. Briefly, LC cells were crosslinked with a 1% formaldehyde solution for 10 min at room temperature, and the reaction was quenched with 125 mM glycine. DNA fragments ranging from 200 to 500 bp in length were obtained via sonication. Then, the lysates were immunoprecipitated with Anti-L-Lactyl-Histone H3 (Lys18) Rabbit mAb-ChIP Grade (PTMBio, China) or the Rabbit mAb IgG Isotype Control (PTMBio, China). The immunoprecipitated DNA was analyzed by PCR, and the ChIP‒PCR primer sequences are shown in Table S2.

**Luciferase assay**

The core promoter of the IRS1 gene (- 2000 to - 200 bp, relative to the transcription start site of IRS1) was synthesized, cloned and inserted into the pGL3-basic firefly luciferase reporter to generate the IRS1 transcription reporter pGL3-IRS1. The pGL3-IRS1 and pRL-TK (as transfection efficiency controls) plasmids were transfected into LC cells subjected to different treatments. Luciferase activity was measured with the Dual Luciferase Assay System (Promega, USA). Renilla luciferase activity was normalized to firefly luciferase activity.

**Seahorse metabolic analysis**

The Agilent Seahorse XF Mitochondrial Stress Test Kit (Agilent, USA) was used for the experiments. Twenty-four hours prior to the experiment, LC cells subjected to different treatments were washed with PBS and digested with trypsin before being counted with a hemocytometer. The cells were seeded into XF96 microplates at a density of 1.5×10^4^ cells/well. Additionally, probes for measuring the oxygen concentration and pH were hydrated with sterile water. Once the cells reached 70–90% confluence, the medium was replaced with XF detection medium via a multichannel pipette, and the cells were incubated for 1 hour at 37 °C in a CO2-free incubator. The oxygen consumption rate (OCR) and extracellular acidification rate (ECAR) were monitored during the sequential injection of oligomycin (1 μM), carbonyl cyanide-4-(trifluoromethoxy) phenylhydrazone (FCCP) (1 μM), and rotenone or antimycin A (0.5 μM). Analysis reports were generated using Wave Desktop software (Agilent, USA).

**Glucose uptake detection**

Cells per well were seeded in a 24-well plate. The growth medium was removed and cells were washed twice with PBS for 5 minutes each time. Subsequently, 1 mL of 20 μM 2-NBDG working solution was added, followed by 60 minutes of incubation at room temperature. Cells were then washed twice with PBS for 5 minutes each time. Fluorescence microscopy was used to observe green fluorescence (Ex/Em: 488/542 nm).

**Membrane and cytoplasmic protein extraction**

Cell Membrane Protein Extraction Kit (Solibao, EX1500) was used for membrane protein extraction. Protein Extraction Buffer A was supplemented with Protein Extraction Buffer B and a mixture of protease inhibitors, thoroughly mixed, and placed on ice for later use. Approximately 5 × 10^6^ cells were harvested, centrifuged, and washed twice with cold PBS. The cells were then resuspended in cold Extraction Buffer A, vortexed briefly, and centrifuged to collect the supernatant. The supernatant was incubated in a 37°C water bath, followed by centrifugation to remove the upper layer. The remaining liquid was dissolved in 30-100 μl of Membrane Protein Solubilization Buffer C to obtain membrane proteins. We performed cytoplasmic extraction using the PARIS™ Kit Protein and RNA Isolation System (Thermofisher, 00679444). Approximately 10^5 cells were collected, washed with pre-chilled PBS, resuspended on ice, and centrifuged to remove PBS. The pellet was then resuspended in 100-500 μl of cold cell fractionation buffer, incubated on ice for 5-10 minutes, and centrifuged at low speed.

**Bioinformatics analysis**

The RNA-sequencing and microarray data of LC tissues were obtained from the TCGA and GEO databases. In total, 4 independent LC datasets (TCGA-LC, GSE14520, GSE84402 and GSE10186) were analyzed in this study. These files were downloaded and analyzed according to standard procedures. GSEA was employed to identify gene sets related to PYCR1 in LC. Gene expression profiles of LC patients were obtained from the TCGA dataset. LC samples were divided into a high-expression group and a low-expression group according to the expression of LC (the top 25% of samples were grouped as high expression, and the bottom 25% of samples were grouped as low expression). The GSEA v3.0 tool was used to explore the distribution of members of the gene sets from the MSigDB database[2]. If most members in a gene set were positively or negatively related to PYCR1 expression, the set was termed associated with PYCR1.

**References**

[1] Milne K, Sun J, Zaal EA, Mowat J, Celie PH, Fish A, et al. A fragment-like approach to PYCR1 inhibition. 2019;29:2626-2631.

[2] Castanza AS, Recla JM, Eby D, Thorvaldsdóttir H, Bult CJ, Mesirov JP. Extending support for mouse data in the Molecular Signatures Database (MSigDB). Nature methods 2023;20:1619-1620.

**
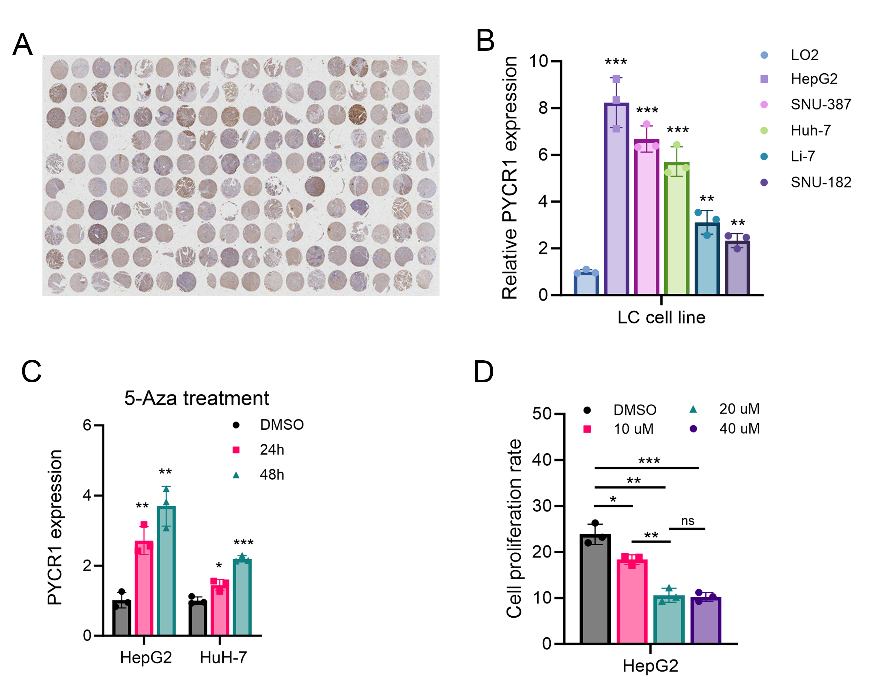
**

**Figure S1. PYCR1 expression is elevated in LC and its high expression is associated with LC progression.** A. Overview of PYCR1 immunohistochemistry results of LC tissue microarray. B. Expression of PYCR1 in normal hepatocytes LO2 and various LC cell lines. C. RNA level of PYCR1 detected in HepG2 and HuH-7 after treating with 5-AZA. D. The proliferation rate of HepG2 cells under treatment with different concentrations of PYCR1 inhibitors. ^*^ *p* < 0.05, ^**^ *p* < 0.01, ^***^ *p* < 0.001.

**
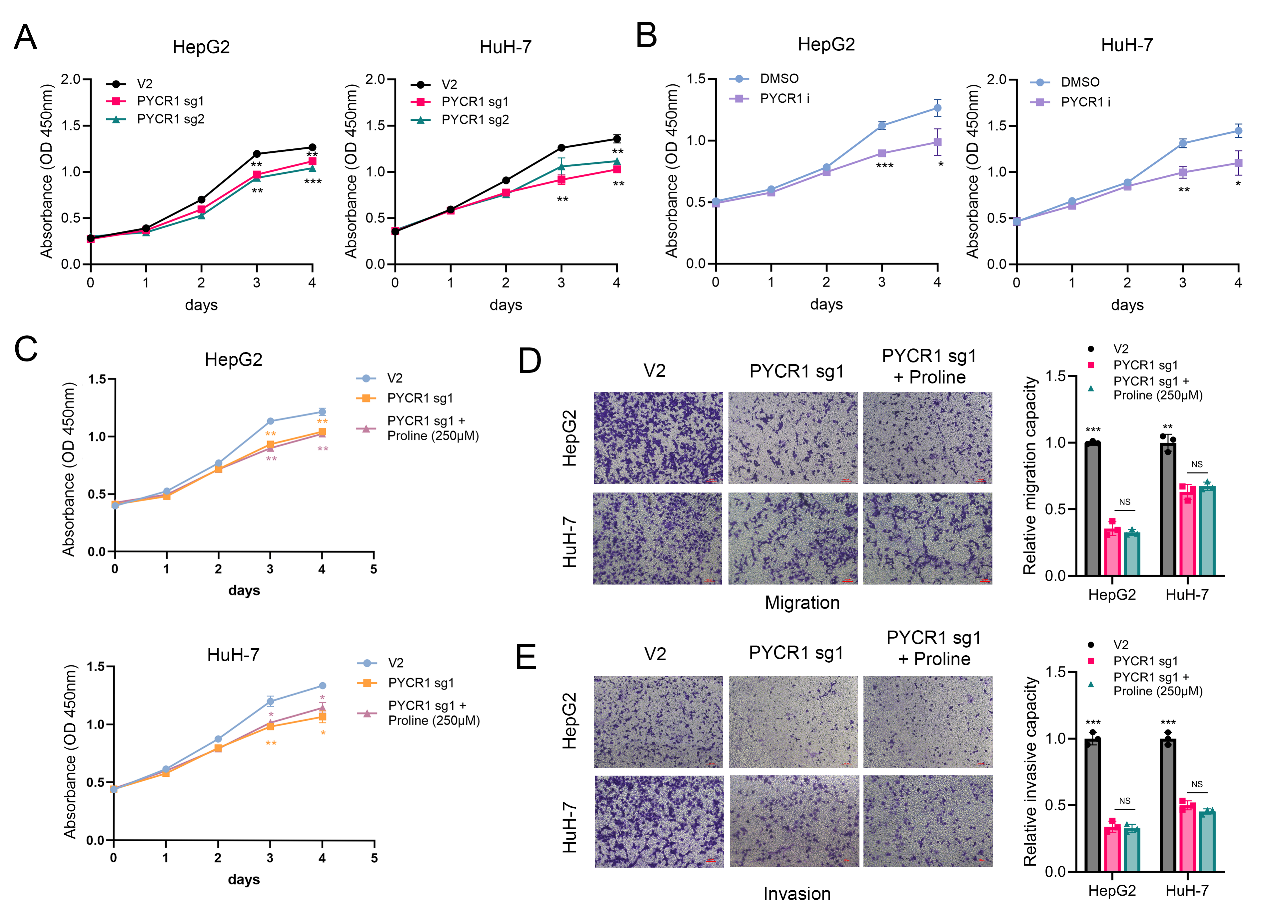
**

**Figure S2. Proline supplementation cannot weaken the effect of knocking out PYCR1 on LC cells.** A, B. HepG2 and HuH-7 cells knocked out or inhibited PYCR1 were subjected to the CCK-8 assay. C. CCK-8 assays were conducted under PYCR1 knocked out in HepG2 and HUH-7 cells with or without proline supplementation. D, E. PYCR1 knocked out HepG2 and HUH-7 cells with or without proline supplementation were subjected to the Transwell migration or invasion assays. ^*^ *p* < 0.05, ^**^ *p* < 0.01, ^***^ *p* < 0.001.


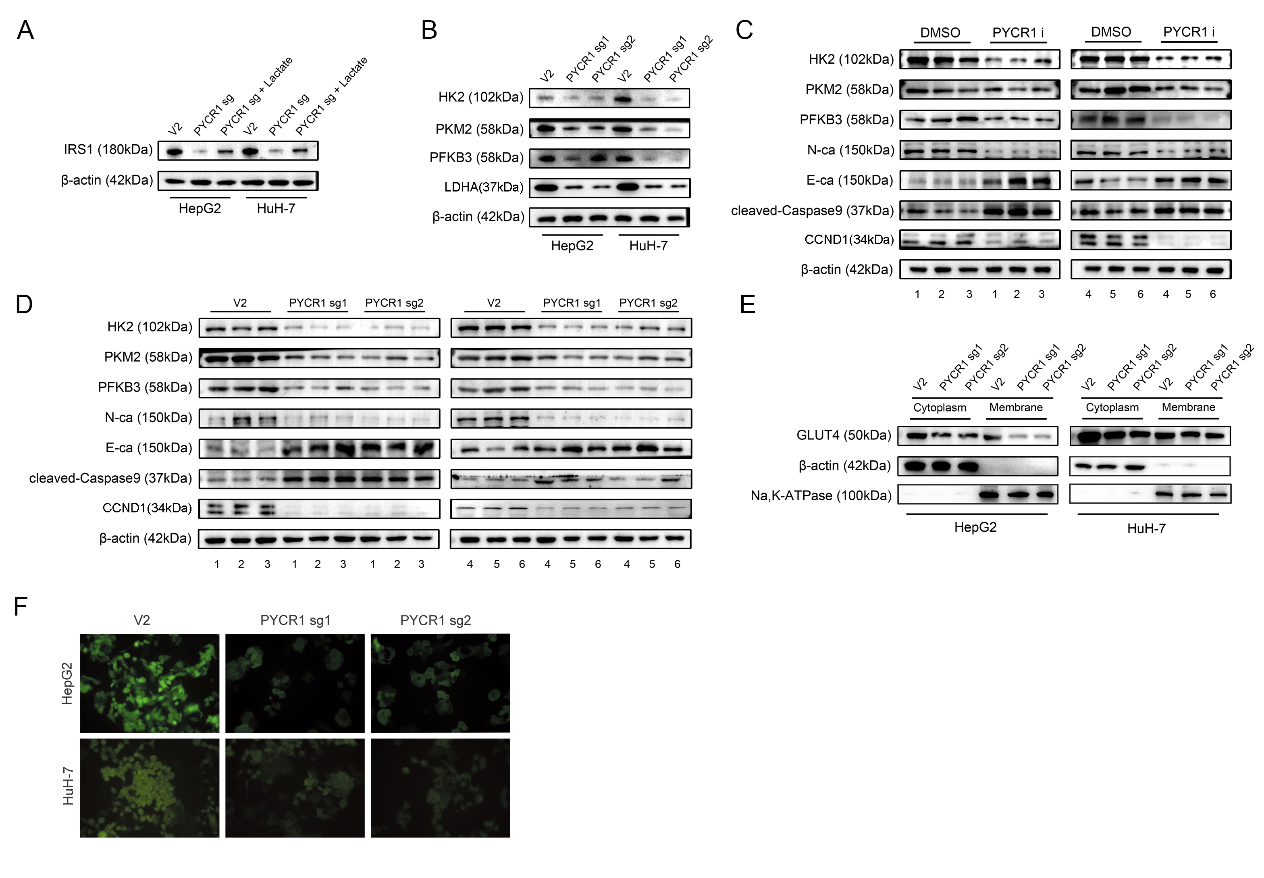


**Figure S3.** **Knockout or inhibition of PYCR1 perturbs glucose-related metabolic processes in LC cells.** A**.** Detection of PYCR1 expression in PYCR1 knockout LC cells after lactate supplementation (15mM 48h). B. The expression of glycolysis-related enzymes was detected after PYCR1 knockout. C, D. Expression of glycolysis-related enzymes and tumor progression associated genes in LC xenograft tissues with PYCR1 knockout or PYCR1 inhibitor treatment. E. Expression of GLUT4 in the cytoplasm and cell membrane after PYCR1 knockout. F. Glucose uptake in LC after PYCR1 knockout.


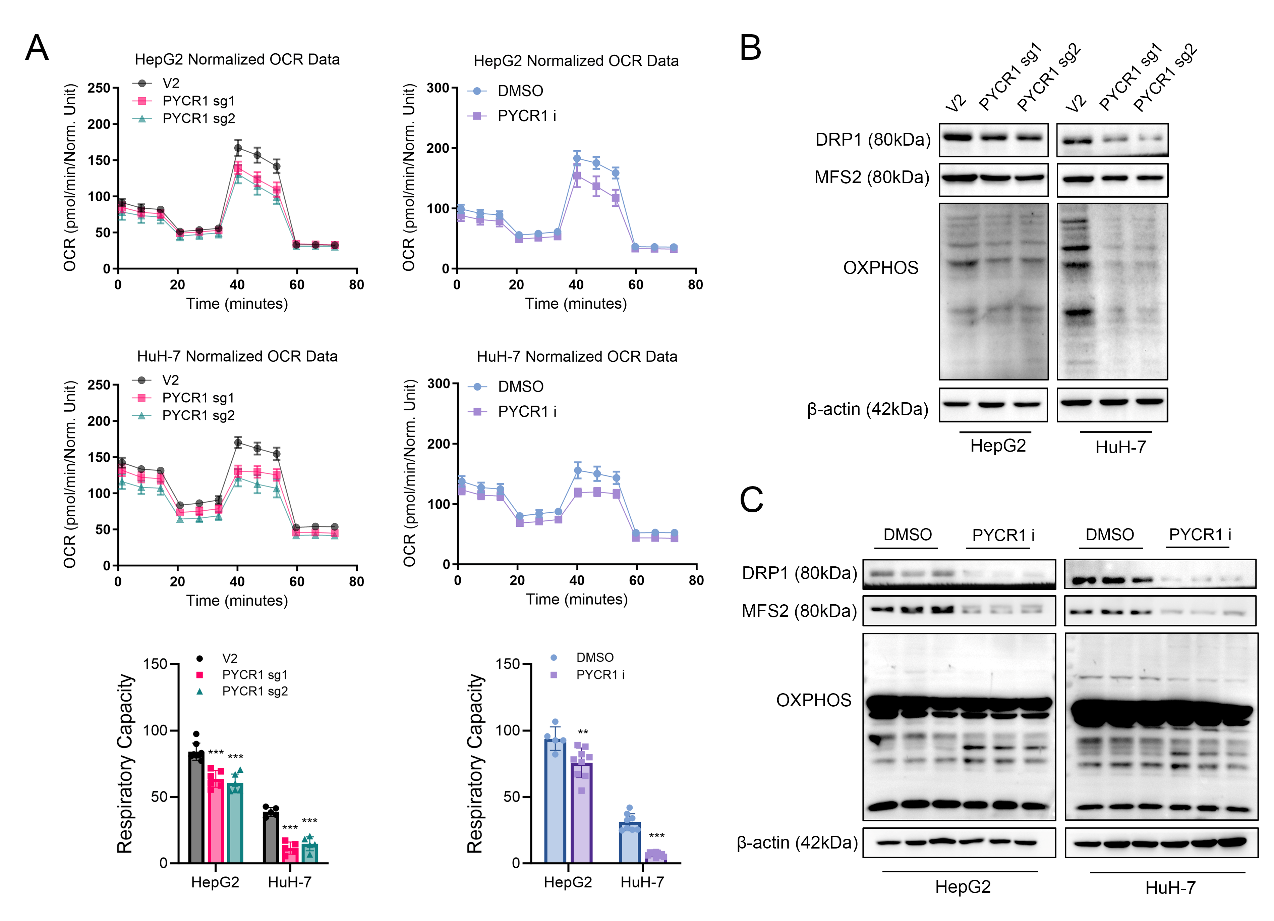


**Figure S4.** **Knockout or inhibition of PYCR1 disrupt mitochondrial function of LC cells.** A**.** The oxygen consumption rate (OCR) was measured in HepG2 and HUH-7 cells after PYCR1 knocked out or inhibited to reflect cellular mitochondrial oxidative phosphorylation level. B, C. Mitochondrial oxidative phosphorylation-related proteins were detected by Western blot in HepG2 and HUH-7 cells after PYCR1 knocked out or inhibited. ^**^ *p* < 0.01, ^***^ *p* < 0.001.

**
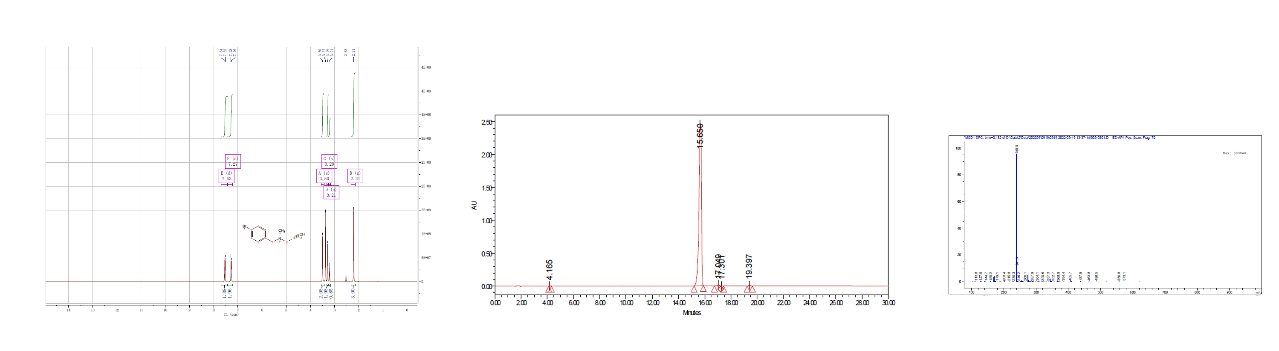
**

**Figure S5.** **Mass spectrometry purity analysis of synthesized PYCR1 inhibitors.**

**Table S1. The clinic-pathological factors of the liver cancer cohort.**

| Characteristics | Number of cases | PYCR1 expression | | P value ^a^ |
| --- | --- | --- | --- | --- |
|  |  | Low (n= 41) | High (n= 42) |  |
| **Gender** |  |  |  |  |
| Female | 18 | 10 | 8 | 0.555 |
| Male | 65 | 31 | 34 |  |
| **Age** |  |  |  |  |
| <60 | 61 | 30 | 31 | 0.947 |
| ≥60 | 22 | 11 | 11 |  |
| **Tumor invasion depth** |  |  |  |  |
| T1 | 54 | 28 | 26 | 0.542 |
| T2 | 29 | 13 | 16 |  |
| **Recurrence** |  |  |  |  |
| non-recurrence:N | 37 | 21 | 16 | 0.229 |
| recurrence:R | 46 | 20 | 26 |  |
| **Pathological grade** |  |  |  |  |
| Ⅰ-Ⅰ~Ⅱ | 47 | 30 | 17 | ***0.003*** |
| Ⅱ-Ⅱ~Ⅲ | 36 | 11 | 25 |  |
| **survival** |  |  |  |  |
| live:0 | 38 | 24 | 14 | ***0.021*** |
| dead:1 | 45 | 17 | 28 |  |

**Table S2： The list of primers.**

| **qPCR primers** |  |  |
| --- | --- | --- |
|  | **Forward Primer** | **Reverse Primer** |
| PYCR1 | TGCCTTGCATGTGCTGGAGAGT | GCTTCACCTTGTCCAGGATGGT |
| IRS1 | AGTCTGTCGTCCAGTAGCACCA | ACTGGAGCCATACTCATCCGAG |
| SOS1 | GAGTGAATCTGCATGTCGGTT | CTCTCATGTTTGGCTCCTACAC |
| SOS2 | CCGCAGCCTTACGAGTTCTTC | GGATGCACTTGTTCCTGAACC |
| ACTB | CATGTACGTTGCTATCCAGGC | CTCCTTAATGTCACGCACGAT |
| **ChIP-PCR primers** | |  |
|  | **Forward Primer** | **Reverse Primer** |
| IRS1-CHIP | GGCTACCCGAAAGCACGA | CCGTGGGTCAGTCTTACCTG |

**Table S3: Information of antibodies.**

|  |  | **Western blot** | **ChIP** | **IHC** |
| --- | --- | --- | --- | --- |
| PYCR1 Polyclonal antibody | proteintech #13108 | 1:1000 |  | 1:200 |
| Cyclin D1 Mouse Monoclonal antibody | proteintech #60186 | 1:2000 |  |  |
| CDK4 (D9G3E) Rabbit mAb | cell signaling technology #12790 | 1:1000 |  |  |
| CDK6 (D4S8S) Rabbit mAb | cell signaling technology #13331 | 1:1000 |  |  |
| Cleaved Caspase-9 (Asp330) (D2D4) Rabbit mAb | cell signaling technology #7237 | 1:1000 |  |  |
| Beta Actin Monoclonal antibody | proteintech #66009 | 1:20000 |  |  |
| Vimentin Rabbit Polyclonal | proteintech #10366 | 1:1000 |  |  |
| E-cadherin Rabbit Polyclonal | proteintech #20874 | 1:1000 |  |  |
| MMP-9 (D6O3H) Rabbit mAb | cell signaling technology #13667 | 1:1000 |  |  |
| N-cadherin Polyclonal antibody | proteintech # 22018 | 1:2000 |  |  |
| IRS1 Polyclonal antibody | proteintech # 17509 | 1:1000 |  | 1:100 |
| SOS1 Antibody | cell signaling technology #5890 | 1:1000 |  |  |
| KI67 Rabbit Polyclonal antibody | proteintech #27309 |  |  | 1:4000 |
| PI3 Kinase p110α (C73F8) Rabbit mAb | cell signaling technology #4249 | 1:1000 |  |  |
| Phospho-PI3 Kinase p85 (Tyr458)/p55 (Tyr199) Antibody | cell signaling technology #4228 | 1:1000 |  |  |
| MEK1/2 Antibody | cell signaling technology #9122 | 1:1000 |  |  |
| Phospho-MEK1/2 (Ser217/221) (41G9) Rabbit mAb | cell signaling technology #9154 | 1:1000 |  |  |
| p44/42 MAPK (Erk1/2) Antibody | cell signaling technology #9102 | 1:1000 |  |  |
| Phospho-p44/42 MAPK (Erk1/2) (Thr202/Tyr204) Antibody | cell signaling technology #9101 | 1:1000 |  |  |
| Akt (pan) (11E7) Rabbit mAb | cell signaling technology #4685 | 1:1000 |  |  |
| Phospho-Akt (Thr308) Antibody | cell signaling technology #9275 | 1:1000 |  |  |
| Phospho-Akt (Ser473) Antibody | cell signaling technology #9271 | 1:1000 |  |  |
| mTOR (7C10) Rabbit mA | cell signaling technology #2983 | 1:1000 |  |  |
| Phospho-mTOR (Ser2448) (D9C2) XP Rabbit mAb | cell signaling technology #5536 | 1:1000 |  |  |
| Anti-L-Lactyl-Histone H3 (Lys18) Rabbit mAb | PTMBIO PTM-1406RM | 1:1000 | 6 μg/ 5×10^6^ cells |  |
| Anti-L-Lactyl Lysine Rabbit mAb | PTMBIO PTM-1401RM | 1:1000 |  |  |
| GLUT1 Polyclonal antibody | Proteintech # 21829-1-AP | 1:1000 |  |  |
| GLUT2 Polyclonal antibody | Proteintech # 20436-1-AP | 1:500 |  |  |
| GLUT4 Monoclonal antibody | Proteintech # 66846-1-Ig | 1:1000 |  |  |
| Na,K-ATPase antibody | Abcam # ab76020 | 1:20000 |  |  |
| PFKFB3 Polyclonal antibody | Proteintech # 13763-1-AP | 1:1500 |  |  |
| Hexokinase 2 Polyclonal antibody | Proteintech #22029-1-AP | 1:5000 |  |  |
| PKM2-specific Polyclonal antibody | Proteintech #15822-1-AP | 1:1000 |  |  |
